# Cancer mortality trends in the Umbria region of Italy 1978–2004: a joinpoint regression analysis

**DOI:** 10.1186/1471-2407-7-10

**Published:** 2007-01-16

**Authors:** Fabrizio Stracci, Antonio Canosa, Liliana Minelli, Anna Maria Petrinelli, Tiziana Cassetti, Carlo Romagnoli, Francesco La Rosa

**Affiliations:** 1 Nominative Registry of Causes of Deaths, Umbrian Population Cancer Registry, Perugia, Italy; 2 Dpt. Medical-Surgical Special. & Public Health, Public Health Section, University of Perugia, Italy; 3 Department of Health, Regional Government of Umbria, Perugia, Italy

## Abstract

**Background:**

The aim of the present paper was to analyse cancer mortality in the Umbria region, from 1978 to 2004. Mortality trends depend on a number of factors including exposures, health system interventions, and possibly artefact (e.g. classification change, variations of data completeness). Descriptive data on mortality only allow for generation of hypotheses to explain observed trends. Some clues on the respective role of possible mortality determinants may be found comparing mortality with incidence and survival data.

**Methods:**

Mortality data for the periods 1978–1993 and 1994–2004 were supplied by the National Institute of Statistics (ISTAT) and the Regional Causes of Death Registry (ReNCaM) respectively. Sex and site-specific mortality time trends were analysed by the "joinpoint regression" method.

**Results:**

For all sites combined, in both sexes, the standardised rate was first increasing before the end of the eighties and decreasing thereafter. Gastric cancer mortality showed a different trend by gender; that is the rate constantly decreased over the period among females while, for males, it was first increasing up to 1985 and decreasing thereafter. Liver cancer trend showed a pattern similar to gastric cancer. Large bowel cancer showed a gender specific trend, that is it was increasing among males and stable among females. Also lung cancer mortality varied by gender: it started to decline after 1989 among males but was steadily increasing over the study period among women. A decreasing trend for female breast cancer mortality began in 1994. Prostate cancer mortality trend is the only one showing two significant joinpoints: mortality decreased up to 1990, then it increased up to 1998 and, finally, was decreasing.

**Conclusion:**

Overall cancer mortality was decreasing in both sexes in Umbria and this favourable trend will probably continue and further improve since population screening against breast, cervix, and large bowel cancers were recently introduced. Besides gastric cancer, tobacco-related cancers and prostate cancer mainly contributed to mortality reduction in males, whereas breast cancer mainly contributed to declining mortality in females.

## Background

The analysis of mortality trends is an important tool to monitor cancer control, and evaluate the outcomes of modifications in population lifestyle, environmental risks, and the effectiveness of health care [1]. Mortality reduction remains the main objective of interventions based on screening and treatment, and the ultimate indicator to assess the effectiveness of most cancer control strategies. Many reports showing significant changes in cancer mortality are published in Italy and other countries [2-4]. When other cancer burden indicators, like incidence and population-based survival are considered together with mortality, it is possible to discuss on the effectiveness of public health strategies and find clues on the respective role of different interventions. Trend based surveillance may be of particular relevance when data are available for geographic areas or population coinciding with the target of specific interventions and with an autonomous level of the health system.

Umbria is a small region in the centre of Italy (825,826 inhabitants at 2001 census). Regional health structures, including two main oncology centres (Perugia and Terni), are easy to reach by all residents Regional Population screening interventions were introduced since 1998 for the prevention of breast and cervical cancer [5,6]; moreover free offering of pap smear tests for cervix cancer preceded by many years population screening. Opportunistic screening activities are likely to be diffused to some extent for skin melanoma, prostate cancer, and large bowel cancer. Screening activities based on digital rectal examination first and later on PSA testing were present in Umbria since the late eighties [7].

A regional cancer registry, the Umbrian Population Cancer Registry (RTUP), was established in the early '90 so that data on incidence, prevalence and survival are available for the period 1994–2002. The Registry also collects regional mortality data from municipal offices and death certificates, and is publishing yearly official general mortality statistics, updated to the previous year.

The aim of the present paper is to analyse the cancer mortality in the Umbria region, from 1978 to 2004. Possible explanations of observed mortality trend will be proposed with some focus on the period 1994–2004 and on the role of health service interventions. Additional information to interpret mortality trends will be derived by comparison with incidence and survival data. Thus mortality will be used as an indicator of cancer control future needs and success of past interventions.

## Methods

Mortality data were supplied by the National Institute of Statistics (ISTAT) until 1993, while, for the following 1994–2004 period, they were supplied by the regional Nominative Causes of Death Registry, ReNCaM, based on the Registry population Offices of the Umbrian municipalities linked with the archives of death certificates collected by the Local Health Districts and afterward utilised by ISTAT. No major or systematic difference seems to exist comparing ISTAT and ReNCaM based mortality data and, since ReNCaM data are available earlier than ISTAT mortality data, they allow the inclusion of more recent years in the analysis [8].

ReNCaM data were not available before 1994. Causes of death were classified according to the X International Classification of Diseases [9]. Cancer sites examined are listed in table 1. For each site we calculated the age-adjusted mortality rates (AADR). Sex and site-specific trends for standardized mortality rates were analysed by "joinpoint regression" [10], using the SEER software [11]. The Umbria population (males + females, 1991 census) was used as standard in the joinpoint analyses, aiming to reduce the bias due to the exceeding difference in age structure; the world standard population was used instead for comparisons with national and international rates (it was not used as the unique standard because it is very different from the aged population of the Region). Mortality trend is approximated since it is described by straight segments but it is allowed to change during the study period (i.e. segments have different slopes). The grid search method allows the detection of segments best describing data. A year when a change in trend is detected over the study period is called a "joinpoint" and significant joinpoints are retained in the final site specific models. The maximum number of joinpoints allowed for each analysis was three. The expected annual percent changes (EAPCs) are reported to describe linear trends by period.

The unreported sites were disregarded because of variability in mortality rates and low number of cases. The definition of head and neck cancer is not standard; we included mouth, tongue, and pharynx sites (C01–C06, C10–C13) and reported separately larynx cancer trend among males. Uterus was considered as a single site, including cervix (C53), corpus (C54) and undefined uterus (C55), since the inaccuracy of death certifications (leading to the assignment of C55 code) was not constant but decreasing over the study period. Incidence (1994–2002) and survival (1994–98) data for Umbrian population were supplied by the regional cancer registry RTUP [12,13]. Survival rates relative to 1978–1982 period, are referred to incident cases resulting from an *ad hoc *survey carried out in the eighties in the Umbria region [14].

## Results

Results of the joinpoint analyses by sex and cancer site, applied to mortality rates spanning from year 1978 to 2004, are reported in table 1.

**Figure 1:**
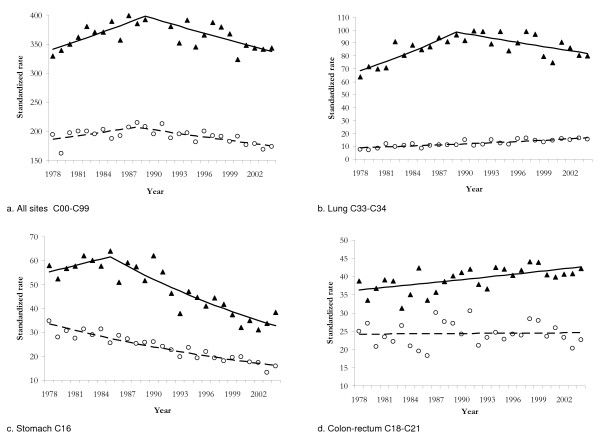
Observed standardized rates per 100.000 inhabitants (▲ males; ○ females) and 'best' joinpoint model estimates (solid line males; dashed line females) for selected cancer sites by gender.

For all sites combined, both in males and females, a significant joinpoint was found (figure 1); moreover the trend shape was similar and the joinpoint year was very close. In males standardised rates significantly increased up to 1989 by 1.41% per year (95% CI from 0.45 to 2.39) and significantly decreased thereafter by 1.10% (95% CI from -1.61 to -0.58); among females the rate increased on average of 1.09% (n.s.) each year till 1988 and afterward decreased of 1.08% per year (p < 0.001).

Gastric cancer showed different mortality trends by gender. It was constantly decreasing among females all over the study period (EAPC = -2.79 P < 0.001) whereas in males it was slightly increasing up to 1985 and significantly decreasing after the year 1985 (EAPC = -3.29, p < 0.02) (figure 1).

Similar trends by gender were found for liver cancer: in females the rates decreased over all the period (EACP = -1.75, p < 0.001); in males a significant joinpoint was located in 1991, the curve was first steeply rising by 5.14% per year and after decreasing by 1.75% per year, with EACPs statistically significant in both periods.

**Figure 2:**
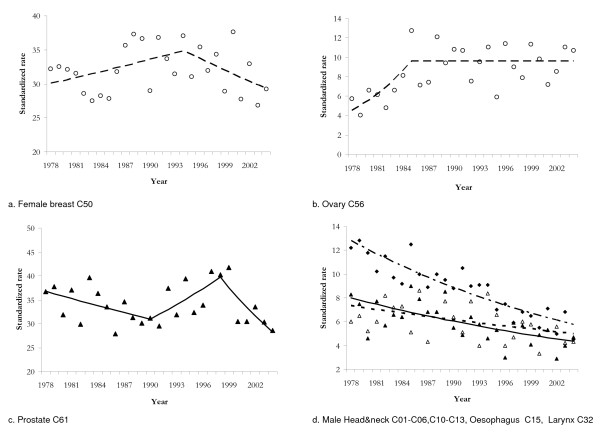
Observed standardized mortality rates per 100.000 inhabitants and 'best' joinpoint model estimates for female breast, ovary, prostate, male head and neck (▲ observed; solid line joinpoint), oesophagus (△ observed; dotted line joinpoint) and larynx cancers (◆ observed; mixed line joinpoint).

Among males, head and neck, oesophagus, and larynx cancers mortality was approximately linearly decreasing over all study period and the slope was significant for all the three sites; the very similar trend observed for the above cancer sites is shown in figure 2.

A significant joinpoint was found for male lung cancer mortality trend in the year 1989; mortality rates were increasing until 1989 at an EACP of 3.36% and decreasing thereafter at an EACP equal to 1.22%. Among women the rates increased steadily over all study period with an EACP equal to 2.38%. All the trends were statistically significant (Fig. 1).

A joinpoint for female breast cancer mortality was detected in 1994: however it is to note that, because of the high variability in mortality rates, none of the slopes is significant (Fig. 2).

Among female cancers, significant joinpoints were found for ovary (Fig. 2) and leukaemias. In both cases, mortality rates were sharply increasing (up to 1985 for ovary and to 1981 for leukaemias), and after remained constant or decreased for ovary and leukaemias respectively. A linearly decreasing trend was observed for mortality from uterine cancer on the whole (EACP = -3.27 p < 0.001).

Prostate cancer mortality trend is the only one showing two significant joinpoints: mortality decreased up to 1990, then it increased up to 1998 and finally decreased (Fig. 2). Only the last slope (EACP = -5.55) was statistically significant (p < 0.02).

Among the considered sites, no-significant trend was revealed for colorectal cancer among females (males faced a constant increase, EAPC = 0.66, p < 0.01) (Fig. 1), for pancreatic and urinary bladder cancer in both sexes, and for leukaemias in males. Finally, for the remaining study sites a significant, approximately linear (i.e. without joinpoints), trend was observed, increasing for melanoma, brain cancer, non Hodgkin lymphomas and myeloma, in both sexes, and decreasing for the other sites.

## Discussion

The present analysis showed a trend toward mortality reduction for many cancer sites. Both in males and in females, a mortality reduction for all cancer sites combined started from 1988–1989. The observed trend is in agreement with national data [2] and data from other countries [15,16]. In the US, mortality rates for all cancer sites combined begun to decrease in 1992–93 [17].

Data from the regional Cancer Registry (1994–2002 period) show increasing incidence rates among females that are mostly due to breast cancer but also to lung and thyroid cancers. Among males a decreasing incidence trend started in the middle nineties, when a decreasing incidence trends for tobacco-related cancers became more marked and also prostate screening activities decreased after initial enthusiasms.

In a previous work, we found evidence for the diffusion of prostate cancer screening in our region by comparing cancer registry's data with data from a population based survey covering the 1978–82 period, and estimated that as many as 45% of the 1994 incident cases may be screen-detected [18]. The opportunistic screening activity probably continued in the following years [7], so that the standardised (world population) incident rate increased slightly from 38.9 to 43.8 per 100.000 inhabitants over the period 1994–2002 (from 25.6 to 33.5 in the 0–74 age group). Mortality rate decreased from 13.6 in 1999 to 9.3 in 2004, and five-year relative survival rate increased from 0.39 for 1978–1982 incident cases, to 0.76 for 1994–1998 incident cases. The prostate cancer opportunistic screening surely changed the meaning and interpretation of incidence (reflecting screening intensity) and survival data. The influence on mortality is less obvious. A peak in mortality rates is uncommon (e.g. Italian mortality is simply decreasing over the period 1988–1999) but is not unique of Umbria [18]. Indeed it is present also in the US data [17,18] and may be associated with changing diagnostic activities since a pronounced increase in mortality occurred earlier in US than in Umbria, and in that country also screening diffusion occurred earlier. Attention devoted to the disease may have influenced health professionals and cause of death coding or a true mortality increase may have occurred. The decreasing mortality trend after peaking may be partly, but not entirely, an artefact due to changes in cause of death coding (i.e. mortality is turning back to the pre-opportunistic screening levels), and partly the consequence of the introduction of effective interventions. Thus mortality reduction may be the result of new cancer treatments or of screening diffusion or both [18,19]; evidence of efficacy for prostate cancer screening, however, is yet inconclusive and also recent studies yielded conflicting results [20-22]. It is also important to note that, if we compare incidence rates as an indicator of screening intensity, PSA screening diffusion in Umbria seems lower than in many other Italian geographic areas covered by cancer registration; indeed highly variable incidence rates among neighbouring Italian areas probably reflect different screening activities [23].

Female breast cancer incidence increased from 66.0 to 83.8 (from 62.7 to 80.8 in the 0–74 age group) and mortality diminished from 19.2 to 15.2 after 1994. The screening, at first opportunistic, became organized for females aged 50–69 starting from 1998–1999. Five-year relative survival improved, for 1994–1998 incident cases, to 86%, if compared to the 71% survival probability reported for 1978–1982 cases. Treatment improvements mostly contributed to mortality reduction because a favourable trend appeared before the introduction of the screening program; further improvements are expected as a consequence of the screening introduction [24].

The joinpoint analysis relative to male lung cancer mortality indicates a decreasing slope after 1989. Really the rates remained constant up to 1997 and after dramatically decreased. This reduction parallel a decrease in male incidence rates (incidence rate decreased from 42.9 in 1994 to 36.2 in 2004). The decline in incidence and mortality rates was predicted many years ago considering the decrease in the number of male smokers and the modification in tar contents of cigarettes smoked [25]; the role of tar content reduction on lung cancer risk, however, has been questioned by more recent research [e.g. [26]]. Unfortunately, despite many efforts and resources devoted, treatment progresses contributed little if any to mortality reduction [2]: in Umbria one-year relative survival rate increased from 0.38 reported for 1978–1982 incident cases to 0.42 for 1994–1998 cases, and five-year survival from 0.11 to 0.13. Lung cancer mortality trend in females showed a constant increase in both mortality and incidence, as in many other European countries, even if some Authors suggest a more favourable trend in European young women over recent calendar years [27].

Over the twenty five years considered, colorectal cancers mortality trend is slowly but significantly increasing in males and stable in females. In Umbria large bowel cancer incidence among males remained steady since 1994, while among females decreased. Five-year survival rates increased from 0.49 to 0.56 (1978–1982 and 1994–1998) in men and from 0.54 to 0.57 in women. This relevant improvement in survival, particularly in males, is likely to be associated with treatment modification [28] rather than with early diagnosis since population screening is just starting in 2006 and opportunistic activities were not very common.

The trend found for gastric cancer mortality is very common in developed countries. In the Umbria Region, twenty five years ago, incidence and mortality rates were very high [14]. The world population age-adjusted incidence rates reached 32.3 per 100,000 inhabitants in males and 16.4 in females. In the Northern areas of the Region, such as in the neighbouring zones of the central Italy, the male mortality rates were close to 50 (i.e. figures close to those reported for Japan and Chile, the countries with the highest mortality in the world).

In 2004 the adjusted mortality rate was 15.8 in men and 6.4 in women and the relative five-year survival increased from 0.25 to 0.33 in males and from 0.28 to 0.32 in females: that is probably due to decreasing prevalence of *Helicobacter pylori *infection, changes in dietary habits [29]. A study carried out in a neighbouring area attributed an increased gastric cancer risk to consumption of a "traditional" diet reach in red and conserved meat [29,30]; a different attention to diet and health could explain the earlier mortality decrease observed among females.

Head and neck, larynx, and oesophagus cancers arise from close topographic areas, share similar histology [31], recognize common risk factors, though the relative importance may vary by site, and show very similar mortality trends (Fig. 2b). All these sites showed a decrease which varied from an EACP equal to -1.46 for oesophagus cancer, to -2.32 for head and neck, and -3.01 for larynx. The incidence trends for these sites were quite the same. Survival rates instead show wide variation among sites [32]. The downward trend may depend perhaps on an earlier stage at diagnosis and more probably on a lower exposure to risk factors like alcohol consumption and tobacco smoking [2].

Liver and pancreas mortality showed different trends. The slopes relative to pancreatic cancer mortality resulted not significant and rates presented a high variability. Incidence rates resulted quite constant from 1994 in males, and decreasing in females. Survival remained very low. An improvement in diagnosis and classification likely affected liver cancer trend [2]; even the joinpoint detected in males, with an EAPC changing in 1991 from 5.14 to -3.10, could be due to this change. As mortality, incidence slowly decreased from first 1990s. Hepatitis B vaccination, decreasing prevalence of HCV infection and some decline in consumption of alcohol beverages may have contributed to the observed trend [2]; indeed, as pointed out above, other alcohol related cancers showed a decreasing mortality trend. The five-year relative survival, close to zero for 1978–1982 cases, was about 10% for 1994–1998 cases. However, it is difficult to assess the role of treatment improvement on survival for liver cancer [28].

All the same, the increasing trend in mortality for brain cancer could be also related to improvement in diagnosis.

Both an improvement in diagnosis and registration, and an increase in risk factors exposure may explain the rising trend in mortality and incidence from melanoma of the skin. Some Authors emphasized the relationship between trends observed in different populations with respect to the promotion of sun protection, level of education and the surveillance of pigmented skin lesions [33,34]. Some early diagnosis activities (e.g. melanoma day initiatives) contributed, in Umbria, to an upward incidence and survival trend. However if compared to other Italian and European areas, relative survival in our Region is rather low, (0.64 at five-year).

The difficulty to consider separately the different uterus sites when analysing mortality, hinders the interpretation of trends. Clearly the diffusion of Pap smear testing for cervix cancer may have led to some improvement in stage at diagnosis but it mostly reduced cancer incidence, by removing premalignant lesions before progression [5].

Both incidence and five-year relative survival rate for cervix and corpus uteri cancers are quit stable. Relative survival for the 1994–1998 patients was 0.64 and 0.79 respectively for cervix and corpus uteri. The relatively low survival from cervix cancer is based on few invasive cancers (about 40 per year), arising in somewhat older women (i.e. they are probably more aggressive cancers diagnosed in women non participating in pap screening) [5]. Ovarian cancer mortality rates showed a very high variability; an improvement in diagnostic definition of abdominal cancers is likely to contribute to the increasing trend observed from 1985 onward.

Joinpoint analysis of urinary bladder cancer evidenced a decreasing trend that is non significant in males and significant in females (EAPC -1.04). The changing criteria for coding non-infiltrating urothelial carcinomas hamper the possibility to evaluate incidence and survival trends [35]; to better analyse bladder cancer trends it would be necessary to consider information about stage at diagnosis and grading.

Different mortality trends were observed for Hodgkin's (significant decrease in both sexes) and non-Hodgkin's (significant increase) lymphomas. These differences are clearly due to a different efficacy of available treatment [13], even if changes in interpretations and classifications of death certificates over time may have influenced results [36]. Indeed five-year relative survival rate for Hodgkin's disease, in our region, exceeded 85% while for non-Hodgkin's lymphomas the rate was close to 60%. Also incidence trends varied by type and sex, and the regional trends were similar to that reported for other Italian areas [23].

The availability of more sensitive diagnostic tools and more accurate reporting of diagnoses may have contributed to the observed increasing trends for more specified sites or cancer types particularly when these trends were paralleled by a decreasing trend for unspecified sites (e.g. mortality for uterus cancer sites). So the raise of mortality from multiple myeloma, in both sexes, may be a consequence of an increased exposure to suspected carcinogens, but also of an improvement in diagnosis; the surprising trend reported for leukaemias, (i.e. non significant in males, with a significant +22 EACP until 1981 and -1 thereafter in females) confirms the difficulty to understand causes of death variations for some cancer sites.

## Conclusion

The analysis of mortality trend is an important tool to monitor cancer control. In Umbria, mortality rates started to decrease slowly but significantly in 1988–89 in both sexes. Among males, declining mortality due to smoking-related cancers and gastric cancer determined a favourable trend. Among females, decreasing mortality seems mainly due to the addition of a decreasing mortality for breast cancer to the secular trend of gastric cancer. Thus, overall mortality reduction is likely to depend on a mix of 'societal' changes (e.g. gastric cancer), preventive efforts (e.g. liver cancer, tobacco-related cancers) and treatment improvement (e.g. breast cancer). The recent introduction of population-based screening interventions against cervix, breast (1998) and colorectal (2006) cancers will probably contribute to reinforce the downward mortality trends over the next years. Declining prostate cancer mortality may be due to early diagnosis, treatment improvement or both. Worrying trends, however, were apparent for lung cancer among females and for other relatively infrequent cancers.

## Competing interests

The author(s) declare that they have no competing interests.

## Authors' contributions

FS performed the statistical analysis and drafted the manuscript.

AC and AMP acquired and codified mortality data.

LM and TC acquired and codified incidence data.

CR coordinated the ReNCaM and RTUP activities.

FLR designed the study and revised the manuscript.

All authors read and approved the final manuscript.

## Pre-publication history

The pre-publication history for this paper can be accessed here:



## Figures and Tables

**Table 1:** Joinpoint analysis by sex and cancer site in the Umbria region, 1978–2004*.

***Site (ICD X)***	***Sex***	***Period***	***EAPC***	***95% Confidence interval***	
All sites C00–C99	M	1978–1989	1.41	0.449	2.387
	M	1989–2004	-1.10	-1.610	-0.581
	F	1978–1988	1.09	-0.049	2.245
	F	1988–2004	-1.08	-1.567	-0.584
Head and neck C01–C06, C10–C13	M	1978–2004	-2.32	-3.368	-1.261
Oesophagus C15	M	1978–2004	-1.46	-2.653	-0.260
Stomach C16	M	1978–1985	1.55	-2.256	5.497
	M	1985–2004	-3.29	-4.085	-2.482
	F	1978–2004	-2.79	-3.193	-2.383
Colon-rectum C18–C21	M	1978–2004	0.66	0.277	0.969
	F	1978.2004	0.07	-0.622	0.758
Liver C22	M	1978–1991	5.14	2.175	8.181
	M	1991–2004	-3.10	-5.342	-0.802
	F	1978–2004	-1.75	-2.469	-1.028
Pancreas C25	M	1978–2004	0.27	-0.610	1.150
	F	1978–2004	0.53	-0.311	1.384
Larynx C32	M	1978–2004	-3.02	-3.024	-2.413
Lung C33–C34	M	1978–1989	3.36	1.811	4.929
	M	1989–2004	-1.22	-2.071	-0.364
	F	1978–2004	2.38	1.718	3.055
Skin melanoma C43	M	1978–2004	3.13	1.153	5.138
	F	1978–2004	2.64	0.841	4.463
Breast C50	F	1978–1994	0.93	-0.256	2.132
	F	1994–2004	-1.72	-3.928	0.533
Uterus C53–C55	F	1978–2004	-3.27	-3.989	-2.553
Ovary C56	F	1978–1985	11.32	1.132	22.524
	F	1985–2004	0.02	-1.574	1.647
Prostate C61	M	1978–1990	-1.41	-3.411	0.626
	M	1990–1998	3.15	-0.794	7.249
	M	1998–2004	-5.55	-9.764	-1.143
Bladder C67	M	1978–2004	0.33	-0.405	1.076
	F	1978–2004	-1.04	-2.089	0.012
Brain C71	M	1978–2004	2.33	1.427	3.234
	F	1978–2004	1.78	0.622	2.954
Hodgkin's disease C81	M	1978–2004	-4.96	-6.947	-2.918
	F	1978–2004	-8.52	-15.980	-0.390
Lymphomas nH C83–C85	M	1978–2004	2.63	1.220	4.066
	F	1978–2004	2.38	0.815	3.976
Multiple myeloma C90	M	1978–2004	1.46	0.069	2.865
	F	1978–2004	2.38	1.356	3.470
Leukaemias C91–C95	M	1978–2004	0.18	-0.178	1.112
	F	1978–1981	22.35	0.333	49.197
	F	1981–2004	-1.01	-1.830	-0.190

* Only significant joinpoints (p < .05) were retained in final models for each site.
